# Supplementary material for: A high-risk retinoblastoma subtype with stemness features, dedifferentiated cone states and neuronal/ganglion cell gene expression
Source: Nat Commun. 2021 Sep 22;12:5578. doi: 10.1038/s41467-021-25792-0 (PMC8458383; doi:10.1038/s41467-021-25792-0)
Supplement: Supplementary file 1 — Supplementary Information [file 41467_2021_25792_MOESM1_ESM.pdf]

Supplementary Information for

A high-risk retinoblastoma subtype with stemness features, dedifferentiated cone states and neuronal/ganglion cell gene expression

Jing Liu[1,2,3,34], Daniela Ottaviani[1,2,4,34], Meriem Sefta[1,2,34], Céline Desbrousses[1,2], Elodie Chapeaublanc[1,2], Rosario Aschero[5], Nanor Sirab[1,2], Fabiana Lubieniecki[5], Gabriela Lamas[5], Laurie Tonon[6], Catherine Dehainault[7,8], Clément Hua[1,2], Paul Fréneaux[7], Sacha Reichman[9], Narjesse Karboul[1,2], Anne Biton[1,2,10,11,30], Liliana Mirabal-Ortega[12,13,14], Magalie Larcher[12,13,14], Céline Brulard[1,2,31], Sandrine Arrufat[7], André Nicolas[7], Nabila Elarouci[3], Tatiana Popova[15], Fariba Némati[16], Didier Decaudin[16], David Gentien[16], Sylvain Baulande[17], Odette Mariani[7], Florent Dufour[1,2], Sylvain Guibert[18], Céline Vallot[18], Livia Lumbroso-Le Rouic[19], Alexandre Matet[19,20], Laurence Desjardins[19], Guillem Pascual-Pasto[21,22], Mariona Suñol[21,23], Jaume Catala-Mora[21,24], Genoveva Correa Llano[21,22], Jérôme Couturier[7], Emmanuel Barillot[10,11], Paula Schaiquevich[5,25], Marion Gauthier-Villars[7,8,15], Dominique Stoppa-Lyonnet[7,8,20], Lisa Golmard[7,8,15], Claude Houdayer[7,8,15,32], Hervé Brisse[26], Isabelle Bernard-Pierrot[1,2], Eric Letouzé[27,28], Alain Viari[6], Simon Saule[12,13,14], Xavier Sastre-Garau[7,33], François Doz[20,29], Angel M. Carcaboso[21,22], Nathalie Cassoux[19,20], Celio Pouponnot[12,13,14], Olivier Goureau[9], Guillermo Chantada[4,21,22,25,35], Aurélien de Reyniès[3,35], Isabelle Aerts[1,2,29,35], François Radvanyi[1,2,35,36*]

[1] Institut Curie, PSL Research University, CNRS, UMR144, Equipe Labellisée Ligue contre le Cancer, 75005 Paris, France
[2] Sorbonne Universités, UPMC Université Paris 06, CNRS, UMR144, 75005 Paris, France
[3] Programme Cartes d'Identité des Tumeurs, Ligue Nationale Contre le Cancer, 75013 Paris, France
[4] Precision Medicine, Hospital J.P. Garrahan, Buenos Aires, Argentina
[5] Pathology Service, Hospital J.P. Garrahan, Buenos Aires, Argentina
[6] Synergie Lyon Cancer, Plateforme de bioinformatique “Gilles Thomas”, Centre Léon Bérard, 69008 Lyon, France.
[7] Département de Biologie des Tumeurs, Institut Curie, 75005 Paris, France
[8] Service de Génétique, Institut Curie, 75005 Paris, France
[9] Institut de la Vision, Sorbonne Université, INSERM, CNRS, 75012 Paris, France
[10] Institut Curie, PSL Research University, INSERM, U900, 75005 Paris, France
[11] Ecole des Mines ParisTech, 77305 Fontainebleau, France
[12] Institut Curie, PSL Research University, CNRS, UMR3347, 91405 Orsay, France
[13] Institut Curie, PSL Research University, INSERM, U1021, 91405 Orsay, France
[14] Université Paris-Saclay, 91405 Orsay, France
[15] Institut Curie, PSL Research University, INSERM U830, 75005 Paris, France
[16] Département de Recherche Translationnelle, Institut Curie, 75005 Paris, France
[17] Institut Curie, PSL Research University, NGS platform, 75005 Paris, France
[18] GeCo Genomics Consulting, Integragen, 91000 Evry, France
[19] Département de Chirurgie, Service d’Ophtalmologie, Institut Curie, 75005 Paris, France
[20] Université de Paris, Paris, France
[21] Institut de Recerca Sant Joan de Déu, 08950 Barcelona, Spain
[22] Pediatric Hematology and Oncology, Hospital Sant Joan de Déu, 08950 Barcelona, Spain
[23] Department of Pathology, Hospital Sant Joan de Déu, 08950 Barcelona, Spain
[24] Department of Ophthalmology, Hospital Sant Joan de Déu, 08950 Barcelona, Spain
[25] National Scientific and Technical Research Council, CONICET, Buenos Aires, Argentina
[26] Département d'Imagerie Médicale, Institut Curie, 75005 Paris, France
[27] Centre de Recherche des Cordeliers, Sorbonne Universités, INSERM, 75006 Paris, France
[28] Functional Genomics of Solid Tumors, équipe labellisée Ligue Contre le Cancer, Université de Paris, Université Paris 13, Paris, France
[29] SIREDO Center (Care, Innovation and Research in Pediatric, Adolescent and Young Adult Oncology), Institut Curie, 75005 Paris, France
[30] Present address: Institut Pasteur – Hub Bioinformatique et Biostatistique – C3BI, USR 3756 IP CNRS – 75015 Paris, France
[31] Present address: INSERM U930, CHU Bretonneau, 37000 Tours, France
[32] Present address: Department of Genetics, Rouen University Hospital, 76000 Rouen, France
[33] Present address: Department of Pathology, Centre Hospitalier Intercommunal de Créteil, 94000 Créteil, France
[34] These authors contributed equally
[35] These authors jointly supervised this work
[36] Lead contact
* Correspondence: francois.radvanyi@curie.fr

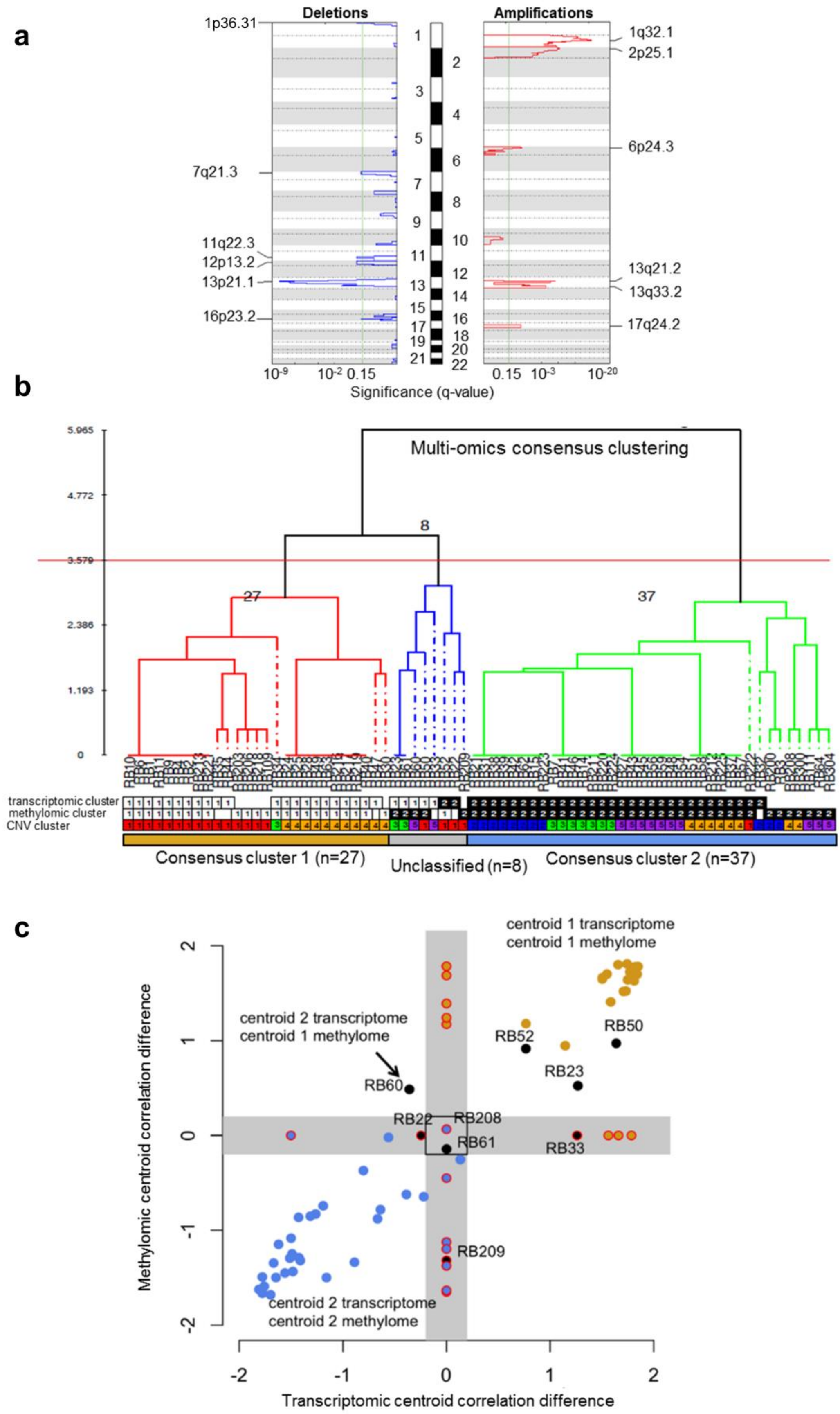

Supplementary Fig. 1 | Multi-omics classification of a series of 72 retinoblastomas.

a, GISTIC plot for copy number alteration data. GISTIC plot of the 72 retinoblastoma specimens, integrating frequency and amplitude to identify significant amplifications (red) and deletions (blue) across the genome (ordered by chromosome). These significant amplifications and deletions were used for copy number alteration data clustering (Fig. 1a).

b, Cluster-of-clusters classification. Cluster-of-clusters analysis for 72 retinoblastoma cases, based on three unsupervised partitions, each partition being obtained using a different genomic platform (transcriptomic, methylomic, and copy number alteration data). A [0;1] normalized co-classification matrix was derived from the 3 initial partitions. Hierarchical clustering was then performed with inter-individual distance defined as (1 – co-classification score) and complete linkage. This analysis identified two major groups of 27 and 37 samples, and one ambiguous/unclassified group of 8 samples. The annotations below the dendrogram represent the clusters of samples defined independently by consensus clustering of all three genomic datasets.

c, Centroid-based classification. This is a schematic representation of the centroid classification methodology used. Each point represents a sample. Samples belonging to cluster-of-clusters 1 are in yellow (n=27), cluster-of-clusters 2 in blue (n=37), and yet unclassified samples in black (n=8). On the X-axis is the difference between each sample's correlation to the cluster-of-clusters 1 transcriptomic centroid and the cluster-of-clusters 2 transcriptomic centroid. The Y-axis represents the same information for the methylomic centroids. Samples with one dataset missing have this difference set to 0 (X coordinate=0 for missing transcriptome, Y coordinate=0 for missing methylome), and the corresponding data points are circled in red. Centroid correlation differences below 0.2 were considered to be outliers, and are in the gray areas. Gray areas therefore contain outliers or samples with one missing dataset. All cluster-of-clusters 1 samples re-classified correctly in methylomic centroid 1 and transcriptomic centroid 1 (or one of the two if a dataset is missing). All but one cluster-of-clusters 2 samples re-classified correctly also. The last one (RB208) was an outlier in the methylomic dataset with no transcriptomic data available, and was thus set to unclassified. For the samples with no cluster-of-clusters attribution (black points): RB52, RB23, RB50 and RB33 were assigned to the first group; RB22, and RB209 were assigned to the second; RB61 was an outlier in both datasets, and RB60 had a discrepancy in the transcriptomic centroid-based classification and the methylomic centroid-based classification, RB61 and RB60 therefore remained unclassified. Ultimately 31 samples were assigned to the first group, 38 to the second, and 3 (RB208, RB60, RB61) remained unclassifed.

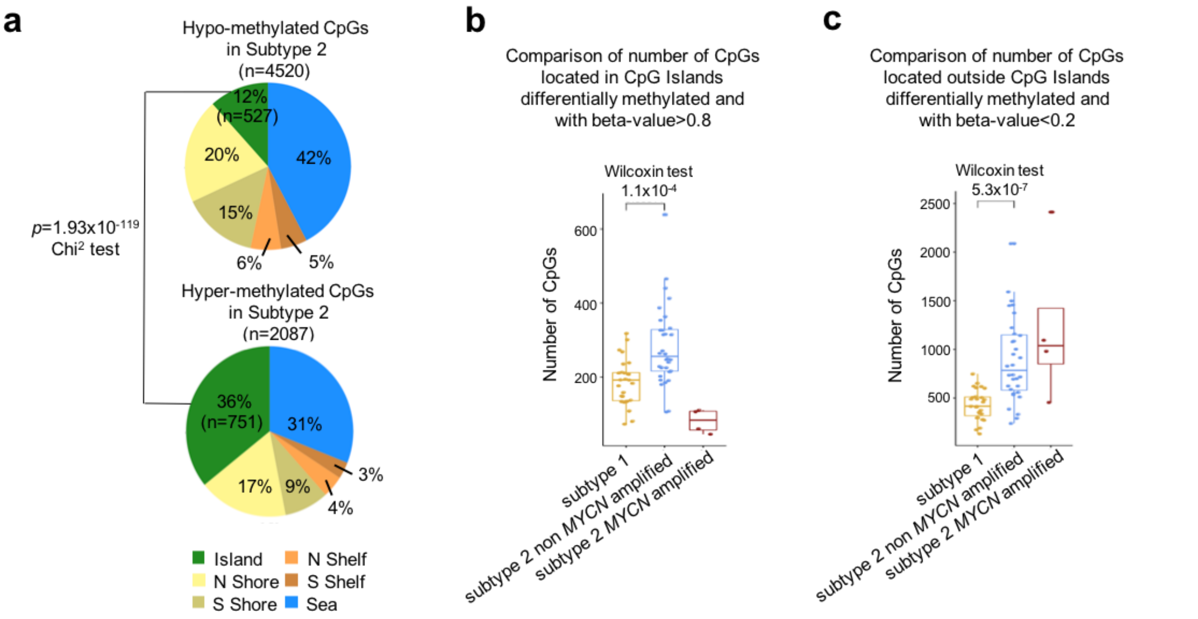

Supplementary Fig. 2 | Distribution of the differentially methylated CpG between the two subtypes in relation to CpG islands.

a, Distribution of the hypomethylated CpGs (upper panel) and the hypermethylated CpGs (lower panel) in subtype 2 as compared to subtype 1, by CpG content and neighborhood context. Hypermethylated CpGs were more frequently located within CpG islands than hypomethylated CpGs (751 of 2087 versus 527 of 4520, p=193x10^{-119}, Chi^2 test) (related to Figure 2F).

b, Boxplot comparing the number of differentially methylated CpGs with high levels of methylation (beta-value within 0.8 to 1) located in CpG Islands in subtype 1 (n=27), subtype 2 non-*MYCN*-amplified (n=32), and subtype 2 *MYCN*-amplified (n=4) tumors (related to Figure 2g upper panel).

c, Boxplot comparing the number of differentially methylated CpGs with low levels of methylation (beta-value within 0 to 0.2) located outsideCpG Islands in subtype 1 (n=27), subtype 2 non-*MYCN*-amplified (n=32), and subtype 2 *MYCN*-amplified (n=4) tumors (related to Figure 2g bottom panel).

b, c, In the boxplots, the central mark indicates the median and the bottom and top edges of the box the 25th and 75th percentiles. Whiskers are the smaller of 1.5 times the interquartile range or the length of the 25th percentiles to the smallest data point or the 75th percentiles to the largest data point. Data points outside the whiskers are outliers. Significance was tested by two-sided Wilcoxon test, p=1.9x10^{-7}.

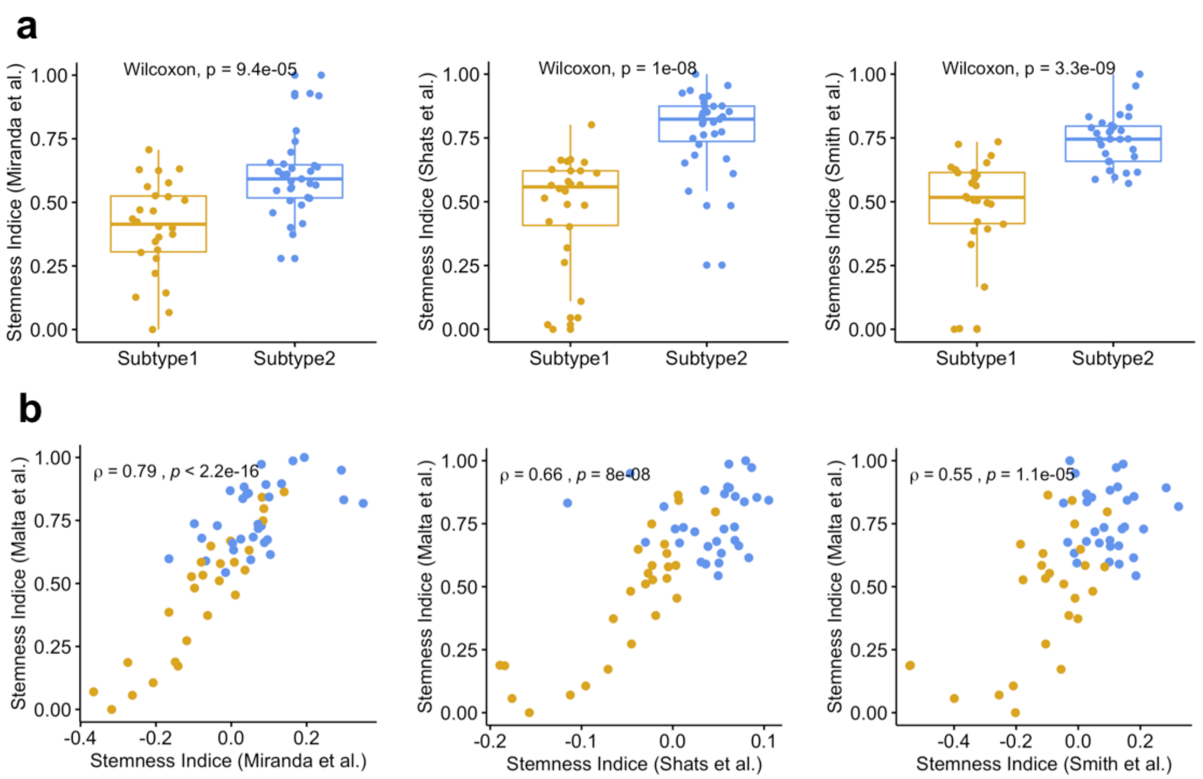

Supplementary Fig. 3 | Stemness Indices estimated by different signatures.

a, Boxplot of stemness indices in the two subtypes of retinoblastoma (subtype 1 tumors: n=26, subtype 2 tumors: n=31) estimated by different signatures (from left to right: Miranda *et al.* 2018[33], Shats *et al.* 2011[35], Smith *et al.* 2018[36]). In the boxplots, the central mark indicates the median and the bottom and top edges of the box the 25th and 75th percentiles. Whiskers are the smaller of 1.5 times the interquartile range or the length of the 25th percentiles to the smallest data point or the 75th percentiles to the largest data point. Data points outside the whiskers are outliers. Data points outside the whiskers are outliers. Statistical tests are two-sided.

b, Scatter plot showing the correlation between stemness indice estimated by Malta et al.’s method (y-axis) or by other signatures (x axis, from left to right: Miranda *et al.* 2018, Shats *et al.* 2011, Smith *et al.* 2018). Two-sided Spearman’s correlation test was applied, rho and p value are shown.

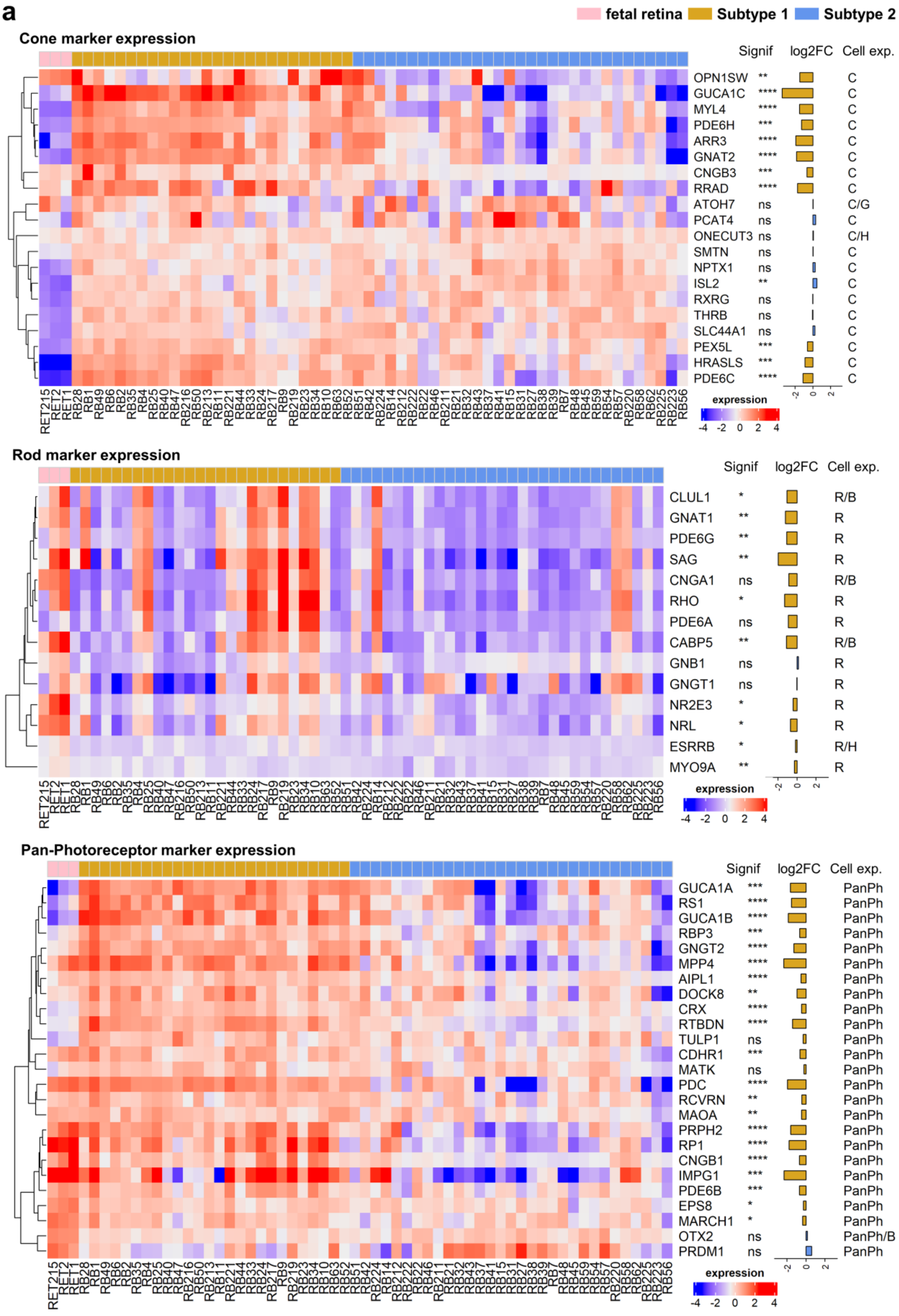
a
fetal retina
Subtype 1
Subtype 2
Cone marker expression
Signif
log2FC
Cell exp.
OPN1SW ** C
GUCA1C **** C
MYL4 **** C
PDE6H *** C
ARR3 **** C
GNAT2 **** C
CNGB3 *** C
RRAD **** C
ATOH7 ns C/G
PCAT4 ns C
ONECUT3 ns C/H
SMTN ns C
NPTX1 ns C
ISL2 ** C
RXRG ns C
THRB ns C
SLC44A1 ns C
PEX5L *** C
HRASLS *** C
PDE6C **** C
expression
Rod marker expression
CLUL1 * R/B
GNAT1 ** R
PDE6G ** R
SAG ** R
CNGA1 ns R/B
RHO * R
PDE6A ns R
CABP5 ** R/B
GNB1 ns R
GNGT1 ns R
NR2E3 * R
NRL * R
ESRRB * R/H
MYO9A ** R
Pan-Photoreceptor marker expression
GUCA1A *** PanPh
RS1 **** PanPh
GUCA1B **** PanPh
RBP3 *** PanPh
GNGT2 **** PanPh
MPP4 **** PanPh
AIPL1 **** PanPh
DOCK8 ** PanPh
CRX **** PanPh
RTBDN **** PanPh
TULP1 ns PanPh
CDHR1 *** PanPh
MATK ns PanPh
PDC **** PanPh
RCVRN ** PanPh
MAOA ** PanPh
PRPH2 **** PanPh
RP1 **** PanPh
CNGB1 **** PanPh
IMPG1 *** PanPh
PDE6B *** PanPh
EPS8 * PanPh
MARCH1 * PanPh
OTX2 ns PanPh/B
PRDM1 ns PanPh

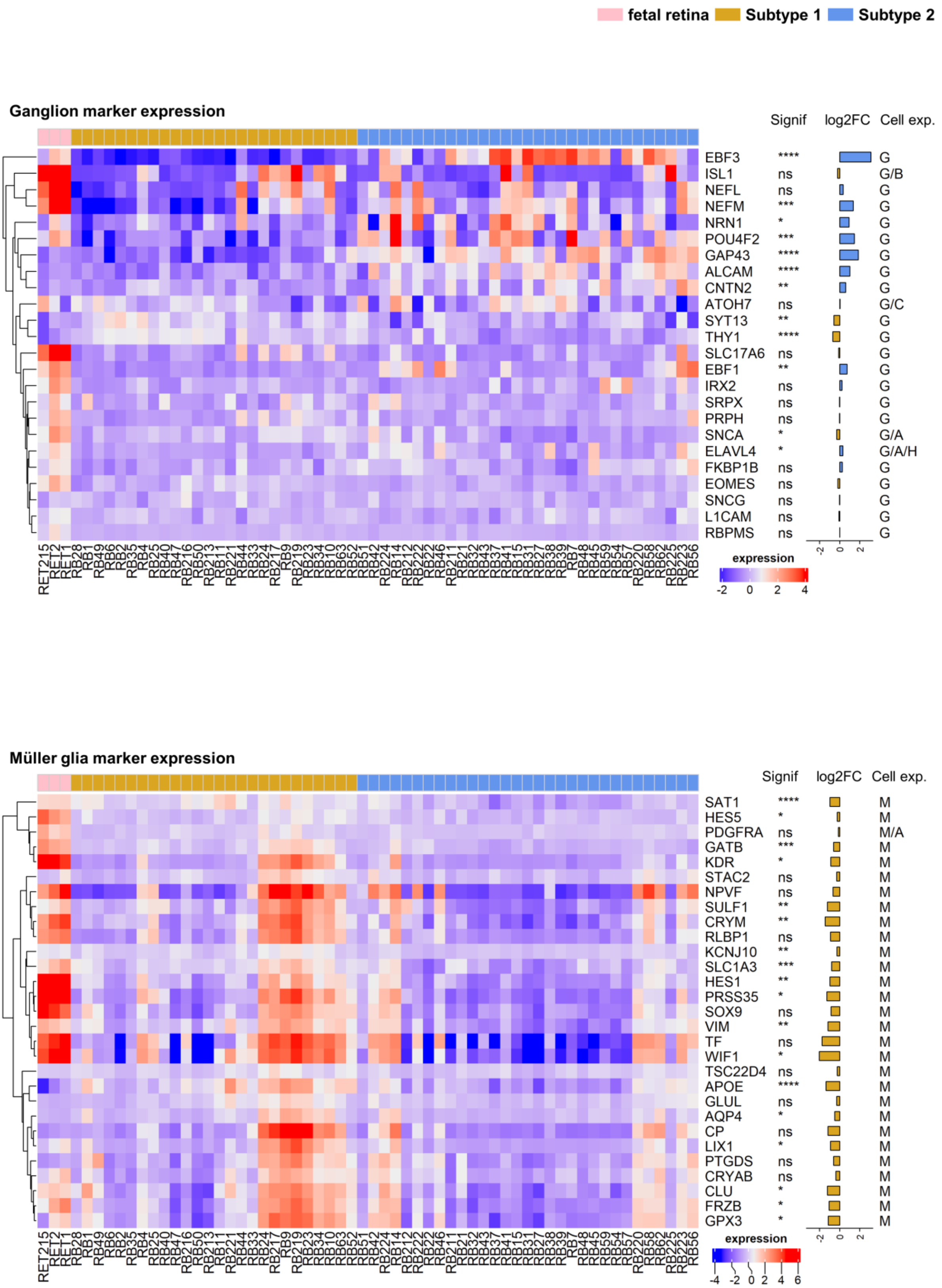
fetal retina
Subtype 1
Subtype 2
Ganglion marker expression
Signif
log2FC
Cell exp.
EBF3 **** G
ISL1 ns G/B
NEFL ns G
NEFM *** G
NRN1 * G
POU4F2 *** G
GAP43 **** G
ALCAM **** G
CNTN2 ** G
ATOH7 ns G/C
SYT13 ** G
THY1 **** G
SLC17A6 ns G
EBF1 ** G
IRX2 ns G
SRPX ns G
PRPH ns G
SNCA * G/A
ELAVL4 * G/A/H
FKBP1B ns G
EOMES ns G
SNCG ns G
L1CAM ns G
RBPMS ns G
expression
-2 0 2 4
-2 0 2
Müller glia marker expression
Signif
log2FC
Cell exp.
SAT1 **** M
HES5 * M
PDGFRA ns M/A
GATB *** M
KDR * M
STAC2 ns M
NPVF ns M
SULF1 ** M
CRYM ** M
RLBP1 ns M
KCNJ10 ** M
SLC1A3 *** M
HES1 ** M
PRSS35 * M
SOX9 ns M
VIM ** M
TF ns M
WIF1 * M
TSC22D4 ns M
APOE **** M
GLUL ns M
AQP4 * M
CP ns M
LIX1 * M
PTGDS ns M
CRYAB ns M
CLU * M
FRZB * M
GPX3 * M
expression
-4 -2 0 2 4 6
-2 0 2

fetal retina Subtype 1 Subtype 2

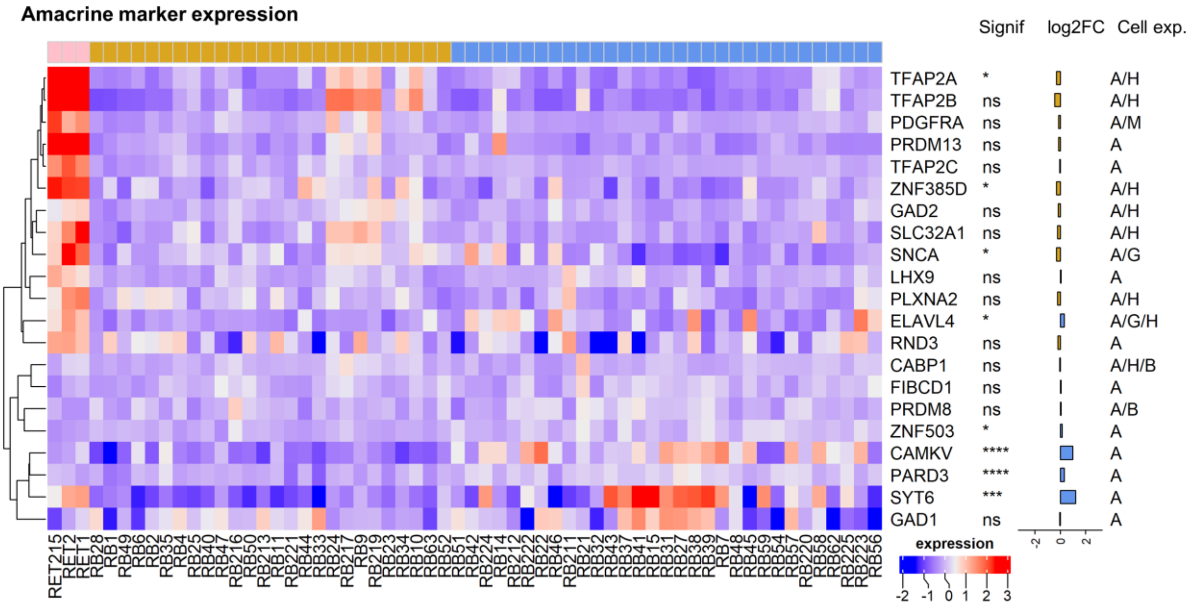

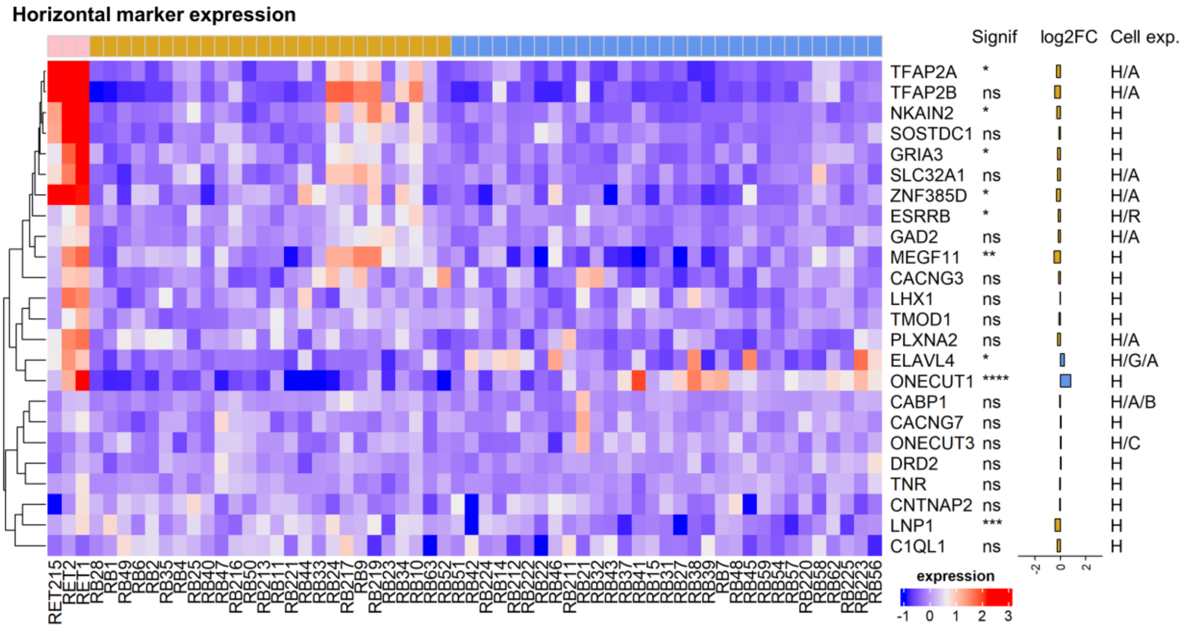

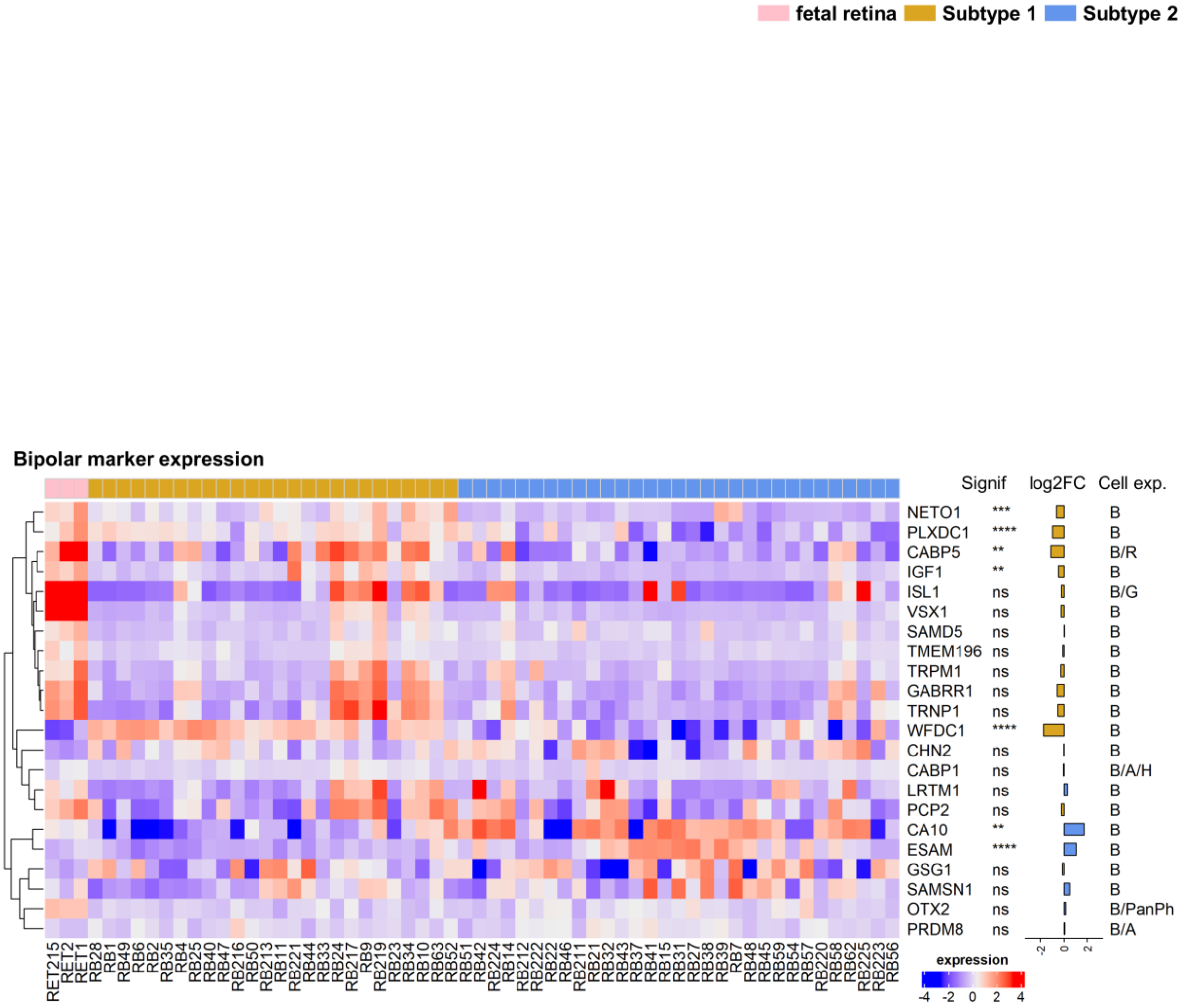

C: Cone
R: Rod
PanPh: Pan-Photoreceptor
G: Ganglion
M: Müller glia
A: Amacrine
H: Horizontal
B: Bipolar

b

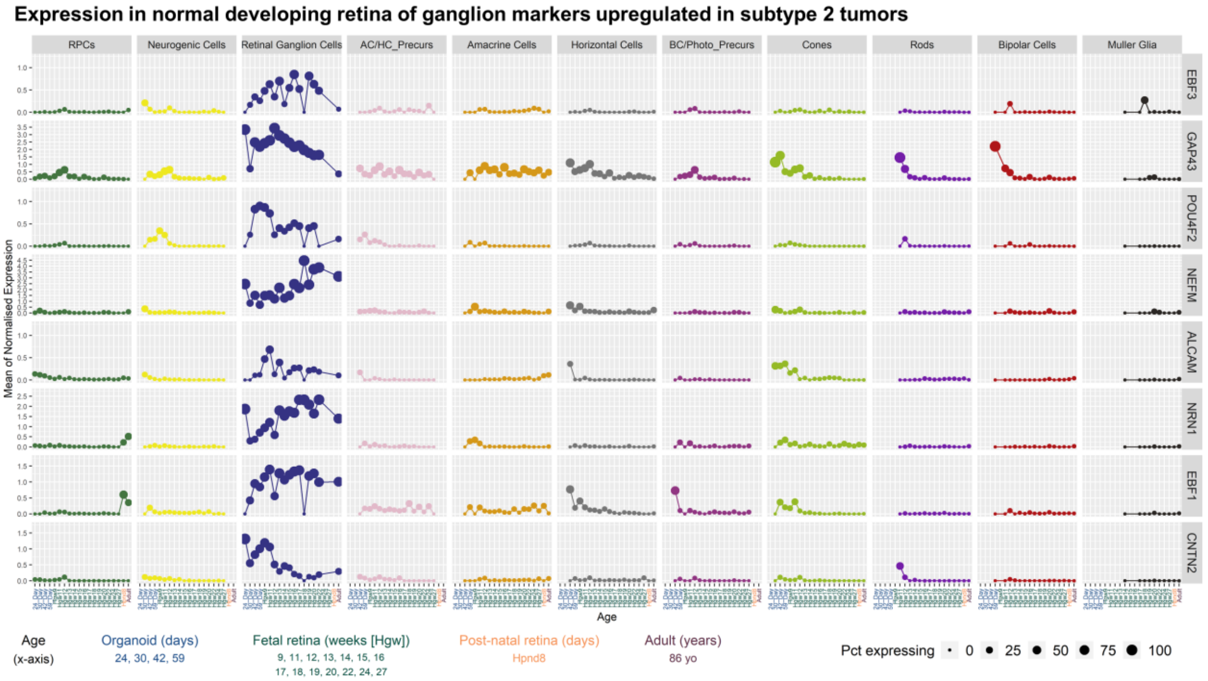

Supplementary Fig. 4 | Retinal cell gene expression analysis in the two subtypes of retinoblastoma.

a, The expression of retinal cell-associated genes is presented as a heatmap, for three fetal retinas, 26 subtype 1 tumors and 31 subtype 2 tumors. A heatmap was constructed for each retinal cell type, indicating the level of expression, the statistical significance (Signif.) and the log2 fold change (log2FC) of expression between subtype 2 and subtype 1 tumors. It is also indicated whether the gene is expressed by more than one retinal cell type (Cell exp.). The complete list of markers is given in Supplementary Table 3. Limma moderated t-test was used for the analysis of gene expression, BH correction was applied, exact p values are provided in Supplementary Table 3.

b, Expression of ganglion markers, displayed in Figure 3e, in the normal developing retina according to cell types (single-cell RNA-seq data from Lu *et al.*, 2020[38]). For each gene and each cell type, a pseudo-dot plot is provided. At each age (x-axis), the dot size is proportional to the percentage of expressing cells (i.e. non-zero counts) and its y-coordinate indicates the mean expression.

For the markers of all cell types, the online tool can be used to visualize their expression:

- cone: https://retinoblastoma-retina-markers.curie.fr/cone,
- rod: https://retinoblastoma-retina-markers.curie.fr/rod,
- pan-photoreceptor: https://retinoblastoma-retina-markers.curie.fr/pan-photoreceptor,
- ganglion: https://retinoblastoma-retina-markers.curie.fr/ganglion,
- muller glia: https://retinoblastoma-retina-markers.curie.fr/muller,
- amacrine: https://retinoblastoma-retina-markers.curie.fr/amacrine,

- horizontal: https://retinoblastoma-retina-markers.curie.fr/horizontal,
- bipolar: https://retinoblastoma-retina-markers.curie.fr/bipolar

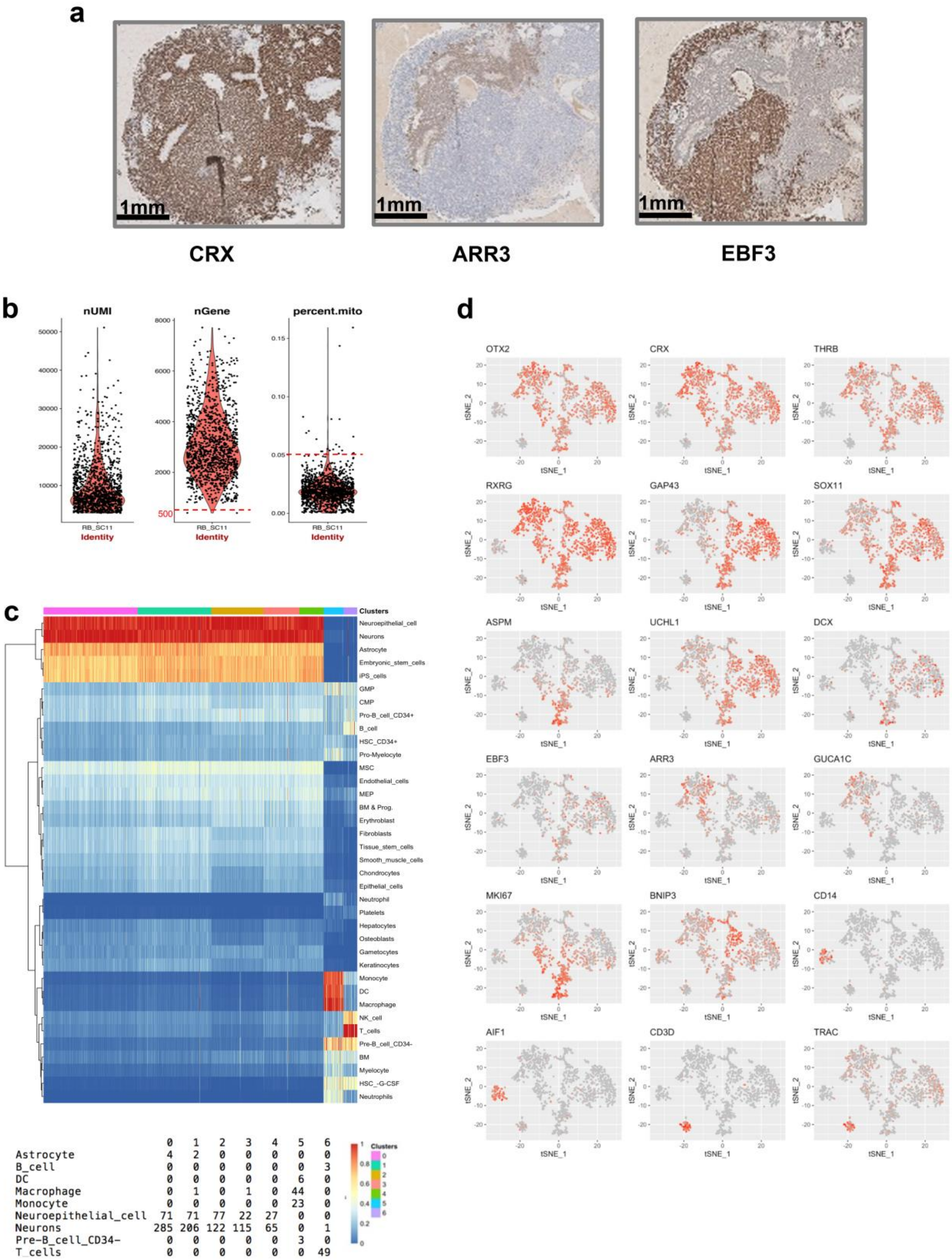

	0	1	2	3	4	5	6
Astrocyte	4	2	0	0	0	0	0
B_cell	0	0	0	0	0	0	3
DC	0	0	0	0	0	6	0
Macrophage	0	1	0	1	0	44	0
Monocyte	0	0	0	0	0	23	0
Neuroepithelial_cell	71	71	77	22	27	0	0
Neurons	285	206	122	115	65	0	1
Pre-B_cell_CD34-	0	0	0	0	0	3	0
T_cells	0	0	0	0	0	0	49

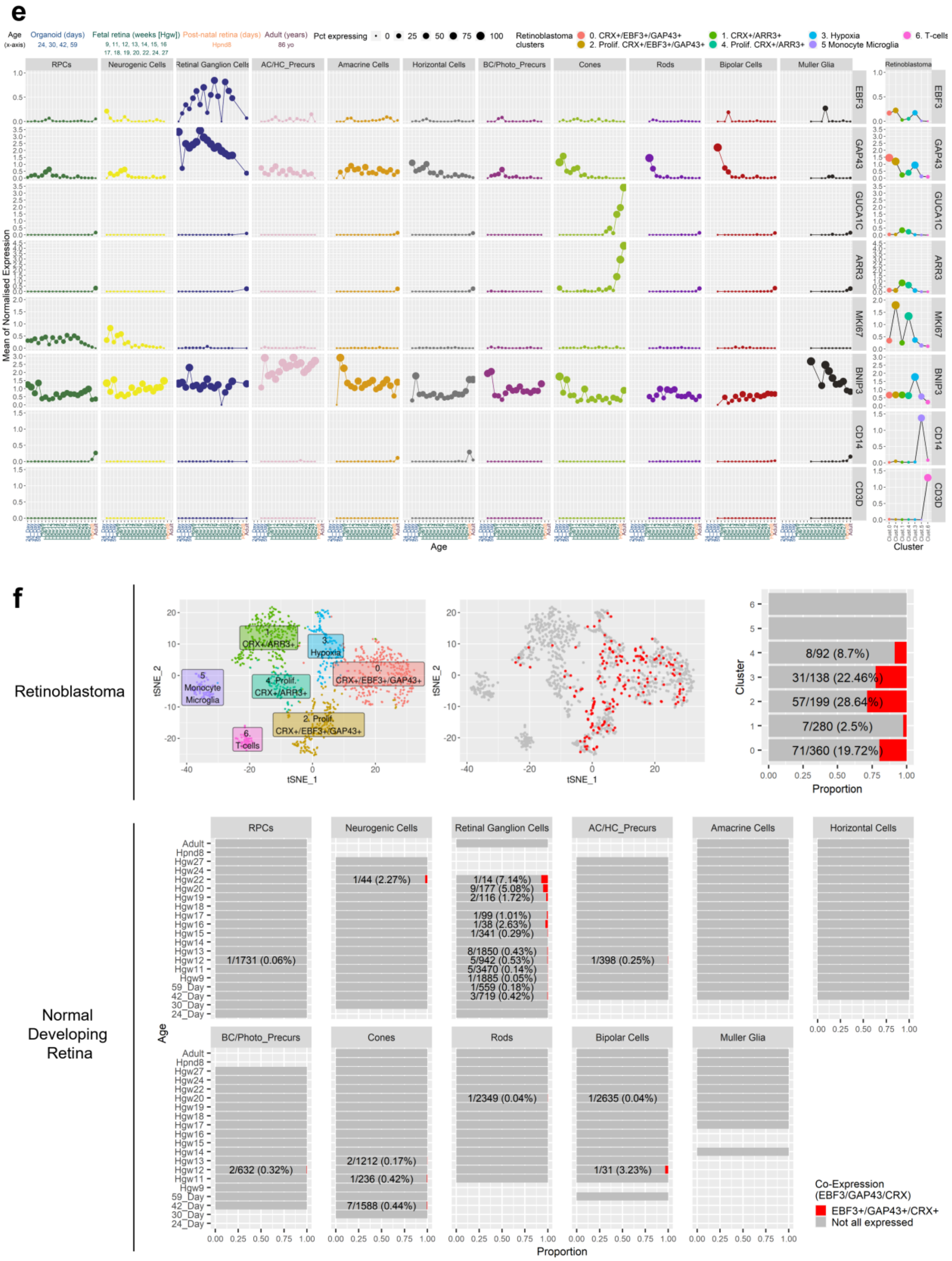

Supplementary Fig. 5 | Data related to single-cell RNA-seq of a subtype 2 retinoblastoma (RBSC11).

a, Immunohistochemical staining of CRX (photoreceptor marker), ARR3 (late cone marker) and EBF3 (ganglion cell marker) for a new case of retinoblastoma (RBSC11).

b, Quality control of single-cell analysis for RBSC11: UMI counts, number of genes and percentage of mitochondrial genes.

c, Heatmap of SingleR annotation scores derived by reference to the HPCA dataset with clusters superimposed for 1198 single cells in RBSC11.

d, Expression of selected retinal/neuronal genes in RBSC11 shown in 2D t-SNE plots: early photoreceptor markers (*OTX2*, *CRX*, *THRB*, *RXRG*); late cone markers (*ARR3*, *GUCA1C*); ganglion/neuronal cell markers (*GAP43*, *SOX11*, *UCHL1*, *DCX*, *EBF3*).

e, Expression of selected genes in the normal developing retina according to cell types (single-cell RNA-seq data from Lu *et al.*, 2020[38]) (left panels). For each gene and each cell type, a pseudo-dot plot is provided. At each age (x-axis), the dot size is proportional to the percentage of expressing cells (i.e. non-zero counts) and its y-coordinate indicates the mean expression. Expression of these genes in the seven cell clusters of retinoblastoma RBSC11 (right panels). For each cluster, the dot size is proportional to the percentage of expressing cells (i.e. non-zero count) and its y-coordinate indicates the mean expression.

The selected genes are representative of the different cell clusters found in tumor RBSC11.

Clusters 0 and 2, expressed early photoreceptor/cone markers (e.g. *CRX*) and neuronal/ganglion cell markers (e.g. *EBF3*, *GAP43*).

Clusters 1 and 4, expressed early photoreceptor/cone markers (e.g. *CRX*) and late cone markers (e.g. *ARR3*).

Clusters 2 and 4 correspond to G2/M cells (expressing *MKI67*)

Cluster 3 corresponds to hypoxic cells (of both tumor cell populations) and expressed *BNIP3*.

Clusters 5 and 6 correspond to normal cells, macrophage/microglia (cluster 5 expressing *CD14*), T-lymphocytes (cluster 6 expressing *CD3D*).

f, Co-expression of *CRX*/*EBF3*/*GAP43* in the retinoblastoma sample (RBSC11) (upper panels) and the normal developing retina (lower panels). The bar plots represent the abundance of the co-expression pattern. When the number of cells displaying co-expression is not zero, the proportion and absolute number of co-expressing cells are displayed. For the retinoblastoma sample, cells co-expressing the three genes (*CRX*/*EBF3*/*GAP43*) are shown in 2D t-SNE plots. The plots can be retrieved from: https://retinoblastoma-retina-markers.curie.fr/coexp-ExtDat.

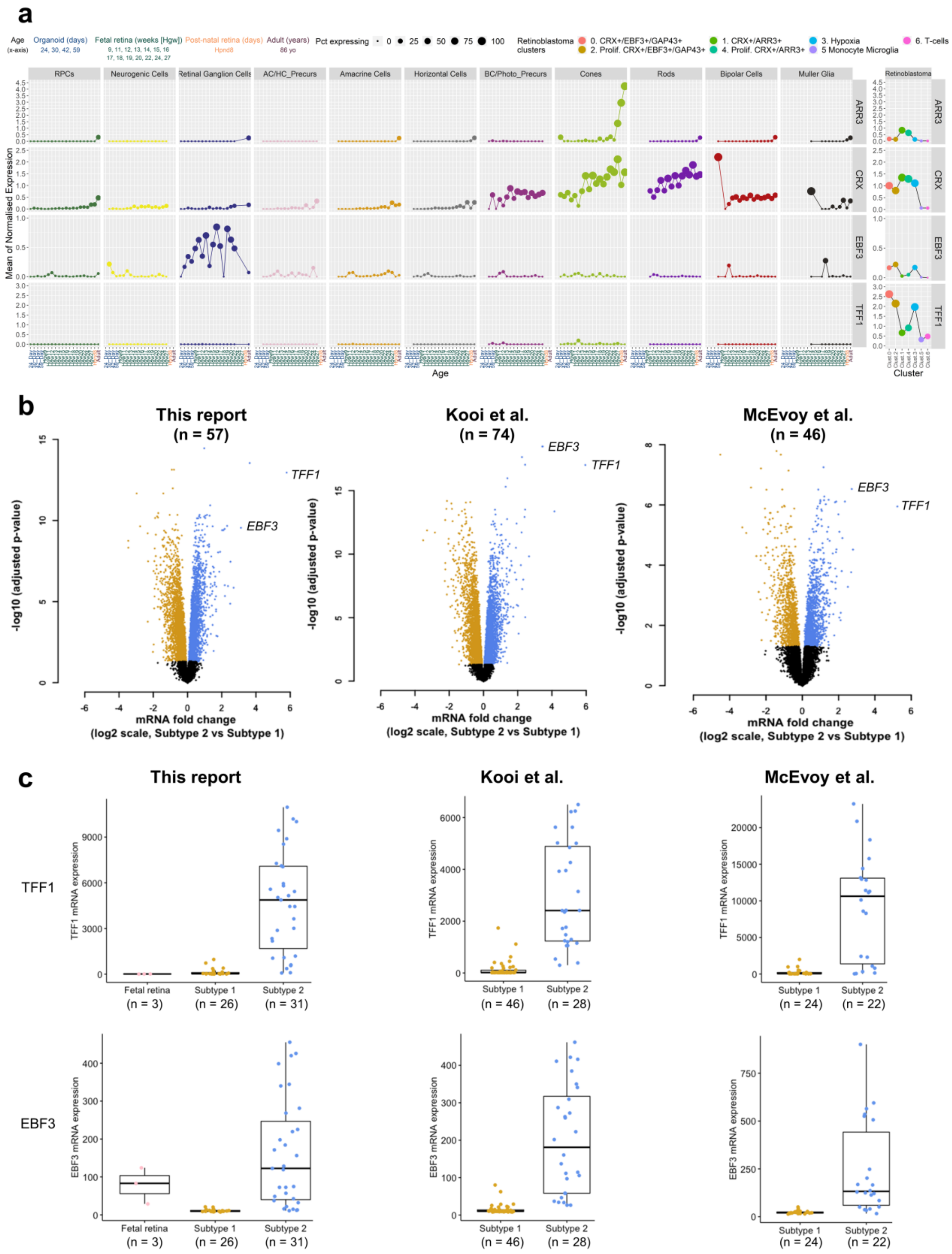

Supplementary Fig. 6 | Expression of *TFF1* and *EBF3* in the normal developing retina and in the two subtypes of retinoblastoma.

a, Expression of TFF1 and EBF3 and of two cone photoreceptor genes (CRX, ARR3) in the normal developing retina according to cell types (single cell RNA-seq data from Lu *et al.*, 2020[38]) (left panels). For each gene and each cell type, a pseudo-dot plot is provided. At each age (x-axis), the dot size is proportional to the percentage of expressing cells (i.e. non-zero counts) and its y-coordinate indicates the mean expression. TFF1 is not expressed in the normal developing retina. Expression of these genes in the seven cell clusters of retinoblastoma RBSC11 (right panels). For each cluster, the dot size is proportional to the percentage of expressing cells (i.e. non-zero count) and its y-coordinate indicates the mean expression.

b, Volcano plots showing that TFF1 and EBF3 are among the most significantly upregulated genes in subtype 2 retinoblastoma (blue) compared to subtype 1 (gold) in our series and in two independent transcriptomic retinoblastoma datasets (Kooi et al.[18], McEvoy et al.[16]). For the subtype assignment of the tumors of these two series, see Methods and Supplementary Fig. 7.

c, Boxplots representing the expression of TFF1 and EBF3 in the two subtypes using three independent datasets (this report, Kooi et al.[18], McEvoy et al.[16]). In the boxplots, the central mark indicates the median and the bottom and top edges of the box the 25th and 75th percentiles. Whiskers are the smaller of 1.5 times the interquartile range or the length of the 25th percentiles to the smallest data point or the 75th percentiles to the largest data point. Data points outside the whiskers are outliers.

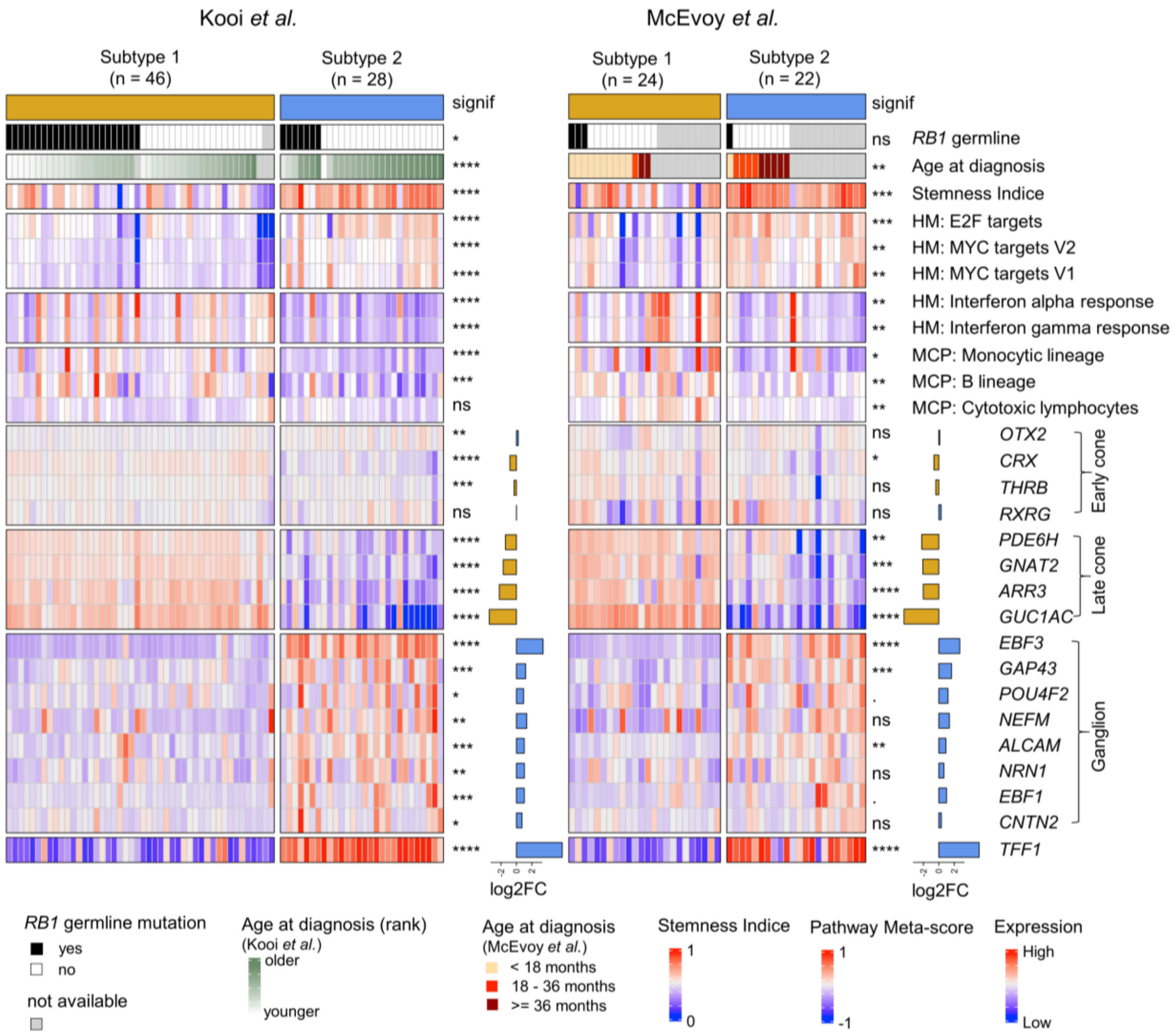

		Kooi et al.			McEvoy et al.		
		Subtype 1 (n = 46)	Subtype 2 (n = 28)	p value	Subtype 1 (n = 24)	Subtype 2 (n = 22)	p value
RB1 germline mutation	yes	23	7	0.02837[a]	3	1	0.6146[a]
	no	21	21		11	9	
Age at diagnosis (rank)	median rank	26	56.5	2.19E-06[b]			
Age at diagnosis	< 18 months				10	1	0.006[c]
	18 - 36 months				1	4	
	>= 36 months				2	5	

Note: a: Fisher's exact test, b Wilcoxon test, c: Chi-square test

Supplementary Fig. 7 | The two retinoblastoma subtypes were different in clinical and molecular features in two additional independent datasets.

The two retinoblastoma subtypes were identified in two additional independent retinoblastoma datasets (Kooi et al. 2015[18], McEvoy et al. 2011[16]) using our centroid-based transcriptomic predictor (see Methods). For each dataset, clinical (*RB1* germline mutation status, age at diagnosis) and molecular (stemness, E2F targets, MYC pathways, interferon responses, estimation of abundance of various immune cells) features, and the expression of cone and

ganglion markers, and of TFF1 were compared between the two subtypes. The features characteristic of each subtype identified in our initial series were found in these two independent series. For germline mutation status and age, the statistical tests used to evaluate the difference between the two subtypes are indicated in the Wilcoxon test was used to evaluate the differences in HALLMARK pathway meta-scores and MCP counter-estimated immune cell abundance between the two subtypes of retinoblastoma; Limma moderated t-test was used for the analysis of gene expression, significance based on adjusted p values is shown. $\log_2$ fold-changes in expression between subtype 2 and subtype 1 are also shown. $p \geq 0.1$ (ns), $p<0.1$ (.), $p<0.05$ (*), $p<0.01$ (**), $p<0.001$ (***), $p<0.0001$ (****).
