# Supplementary material for: A high-risk retinoblastoma subtype with stemness features, dedifferentiated cone states and neuronal/ganglion cell gene expression
Source: Nat Commun. 2021 Sep 22;12:5578. doi: 10.1038/s41467-021-25792-0 (PMC8458383; doi:10.1038/s41467-021-25792-0)
Supplement: Supplementary file 3 — Description of Additional Supplementary Files [file 41467_2021_25792_MOESM3_ESM.pdf]

Description of Additional Supplementary Files

File Name: Supplementary Data 1
Description: Patient information, molecular data types available, molecular subtype assignment

File Name: Supplementary Data 2
Description: Genomic characterization, somatic mutational landscape and DNA methylation profile of retinoblastoma subtypes.

File Name: Supplementary Data 3
Description: Related to Figure 3. Differential gene expression analyses between retinoblastoma subtypes

File Name: Supplementary Data 4
Description: Late stage of cone differentiation in subtype 1 tumors and various stages of cone differentiation in subtype 2 tumors together with the expression of ganglion markers

File Name: Supplementary Data 5
Description: Single-cell transcriptomic analysis

File Name: Supplementary Data 6
Description: Independent series of metastatic retinoblastoma patients (n=19), and of a control group (n=93). Clinicopathological characteristics and immunohistochemical TFF1 data.

File Name: Supplementary Data 7
Description: List of primers used
