# Supplementary material for: A high-risk retinoblastoma subtype with stemness features, dedifferentiated cone states and neuronal/ganglion cell gene expression
Source: Nat Commun. 2021 Sep 22;12:5578. doi: 10.1038/s41467-021-25792-0 (PMC8458383; doi:10.1038/s41467-021-25792-0)
Supplement: Supplementary file 11 — Reporting Summary [file 41467_2021_25792_MOESM11_ESM.pdf]

nature research

Corresponding author(s): Francois RADVANYI

Last updated by author(s): Jul 23, 2021

Reporting Summary

Nature Research wishes to improve the reproducibility of the work that we publish. This form provides structure for consistency and transparency in reporting. For further information on Nature Research policies, see our Editorial Policies and the Editorial Policy Checklist.

Statistics

For all statistical analyses, confirm that the following items are present in the figure legend, table legend, main text, or Methods section.

n/a	Confirmed	
☐	☒	The exact sample size (*n*) for each experimental group/condition, given as a discrete number and unit of measurement
☐	☒	A statement on whether measurements were taken from distinct samples or whether the same sample was measured repeatedly
☐	☒	The statistical test(s) used AND whether they are one- or two-sided<br>*Only common tests should be described solely by name; describe more complex techniques in the Methods section.*
☐	☒	A description of all covariates tested
☐	☒	A description of any assumptions or corrections, such as tests of normality and adjustment for multiple comparisons
☐	☒	A full description of the statistical parameters including central tendency (e.g. means) or other basic estimates (e.g. regression coefficient) AND variation (e.g. standard deviation) or associated estimates of uncertainty (e.g. confidence intervals)
☐	☒	For null hypothesis testing, the test statistic (e.g. *F*, *t*, *r*) with confidence intervals, effect sizes, degrees of freedom and *P* value noted<br>*Give P values as exact values whenever suitable.*
☒	☐	For Bayesian analysis, information on the choice of priors and Markov chain Monte Carlo settings
☐	☒	For hierarchical and complex designs, identification of the appropriate level for tests and full reporting of outcomes
☐	☒	Estimates of effect sizes (e.g. Cohen's *d*, Pearson's *r*), indicating how they were calculated

Our web collection on statistics for biologists contains articles on many of the points above.

Software and code

Policy information about availability of computer code

Data collection	No software was used to collect data.
Data analysis	R >3.2<br>sva 3.30.1 (Leek et al. 2019)<br>MineICA 1.24.0 (Biton 2019)<br>minfi 1.28.4 (Aryee et al. 2014)<br>limma 3.40.6 (Ritchie et al. 2015)<br>clusterProfiler 3.12.0 (Yu et al. 2012)<br>EnrichmentMap 3.2.1 (Merico et al. 2016)<br>Cytoscape 3.7<br>nSolver NanoString 4.0<br>ComplexHeatmap 2.1.1 (Gu Z et al. 2016)<br>ape 5.3 (Desper and Gascuel 2002)<br>CellRanger 2.1.1 (10X Genomics)<br>Seurat 2.3.4 (Butler et al. 2018)<br>SingleR 1.0.1 (Aran et al. 2019)<br>infercnv 0.8.2<br>ggpubr 0.2.0<br>shiny 1.6.0<br>shinydashboard 0.7.1<br>cowplot 1.1.1<br>tidyverse 1.3.0

PyroMark Assay Design software 2.0.2
PyroMark Q24 software v2.0.6.20
Sequencher® v 5.4.1 sequence analysis software (Sanger sequencing)
GISTIC2.0 2.0.22
GLAD algorithm 2.28.1
Genome Alteration Print method (no versioning, scripts available at http://bioinfo-out.curie.fr/projects/snp_gap/)
Bioconductor package DNAcopy 1.50.1
Burrows–Wheeler Aligner (BWA) 0.7.4
MuTect 1.1.5
SomaticIndelDetector 2.1-8
VarScan 2.3.7
Annovar v2014Mar10
Integrative Genomics Viewer 2.3.34
Scripts to reproduces analysis and figures (https://github.com/irisjingliu/RBsubtyping)
Scripts for visualisation tool (https://github.com/ClmtHua/retinoblastoma-retina-markers)

For manuscripts utilizing custom algorithms or software that are central to the research but not yet described in published literature, software must be made available to editors and reviewers. We strongly encourage code deposition in a community repository (e.g. GitHub). See the Nature Research guidelines for submitting code & software for further information.

Data

Policy information about availability of data

All manuscripts must include a data availability statement. This statement should provide the following information, where applicable:

- Accession codes, unique identifiers, or web links for publicly available datasets
- A list of figures that have associated raw data
- A description of any restrictions on data availability

The raw array data are deposited in the Gene Expression Omnibus (GEO) database under accession code GSE58785 [https://www.ncbi.nlm.nih.gov/geo/query/acc.cgi?acc=GSE58785]. The raw whole-exome sequencing data are deposited in the European Genome-Pheome Archive (EGA) database under accession code EGAS00001005248 [https://ega-archive.org/studies/EGAS00001005248]. The raw targeted sequencing data are deposited in the EGA database under accession code EGAS00001005550 [https://ega-archive.org/studies/EGAS00001005550]. The raw single-cell RNA sequencing data are deposited in the EGA database under accession code EGAS00001005178 [https://ega-archive.org/studies/EGAS00001005178]. Data in EGA is available under restricted access, access can be obtained by contacting Retinoblastoma Data Access Committee – Institut Curie (data.office@curie.fr). The public retinoblastoma transcriptomic data used in this study are available in the GEO database under accession code GSE29683 [https://www.ncbi.nlm.nih.gov/geo/query/acc.cgi?acc=GSE29683], GSE59983 [https://www.ncbi.nlm.nih.gov/geo/query/acc.cgi?acc=GSE59983]. The public human developing retina scRNA-seq data used in this study are available in the GEO database under accession code GSE138002 [https://www.ncbi.nlm.nih.gov/geo/query/acc.cgi?acc=GSE138002]. The remaining data are available within the Article, Supplementary Information or Source Data file. Additional data enquiry can be addressed to the Lead contact: francois.radvanyi@curie.fr.

Field-specific reporting

Please select the one below that is the best fit for your research. If you are not sure, read the appropriate sections before making your selection.

☒ Life sciences ☐ Behavioural & social sciences ☐ Ecological, evolutionary & environmental sciences

For a reference copy of the document with all sections, see nature.com/documents/nr-reporting-summary-flat.pdf

Life sciences study design

All studies must disclose on these points even when the disclosure is negative.

Sample size

We included a first series of 102 retinoblastomas studied by genomics, transcriptomics, and methylomics. The sample size represents the largest cohort of retinoblastoma analyzed by multi-omics approaches to date.
We also included an independent series of 112 retinoblastoma with HRPFs (High-Risk Pathological Features), of which 19 metastasized and 93 did not. Considering the low incidence of retinoblastoma and low availability of metastatic cases, the size of the series with 19 metastatic tumors can be considered large.
We studied only one subtype 2 retinoblastoma case by single-cell RNA-seq but this sample was particularly informative, as it was heterogeneous, with one cell population expressing differentiated cone markers and the other expressing neuronal/ganglion cell markers. This case is representative of 30% of type 2 retinoblastoma, as assessed by immunohistochemistry.
In our transcriptomic analysis of retinal markers in retinoblastoma, we included three fetal retinas (20, 23, 27 gestational weeks) for comparison. Our work was also largely based on a large single-cell RNA-seq study of the developing retina (Lu et al., 2020).

Data exclusions

No data were excluded.

Replication

To confirm the identified non-synonymous somatic mutations by WES, we performed a mutation-validation experiment using Sanger sequencing, which yielded a 92% validation rate of variants with allele fractions >10% (100 variants tested). We also confirmed all the mutations identified by targeted high-throughput sequencing by Sanger sequencing.
The expression of key genes was confirmed by both Microarray and NanoString.
Each immunohistochemistry slide was examined by two to three specialists.
Several clinical and molecular properties of the two subtypes were validated in two independent transcriptomic retinoblastoma series (McEvoy et al., 2011; Kooi et al., 2015), including the overexpression of TFF1 and EBF3 in subtype 2 tumors. The heterogeneity of subtype 2

tumors identified by immunohistochemistry was confirmed and further defined on a representative tumor by single cell RNA-seq. The different stages of cone differentiation in retinoblastoma observed in the transcriptomic data were confirmed by comparing retinoblastoma with retinal organoids and the developing retina.

Randomization	This was a retrospective study. This study identifies two molecular subtypes and depicts their molecular and clinical landscapes. Treatment decisions were made independently of this study. Randomization is not applicable.
Blinding	This was a retrospective study. This study identifies two molecular subtypes and depicts their molecular and clinical landscapes. The investigators were blinded to the clinical characteristics at the stage of identifying molecular subtypes.<br>For the Immunohistochemistry experiments, including the study of the metastatic series, investigators were blinded to the molecular subtypes.

Reporting for specific materials, systems and methods

We require information from authors about some types of materials, experimental systems and methods used in many studies. Here, indicate whether each material, system or method listed is relevant to your study. If you are not sure if a list item applies to your research, read the appropriate section before selecting a response.

Materials & experimental systems

n/a	Involved in the study
☐	☒ Antibodies
☐	☒ Eukaryotic cell lines
☒	☐ Palaeontology and archaeology
☒	☐ Animals and other organisms
☐	☒ Human research participants
☒	☐ Clinical data
☒	☐ Dual use research of concern

Methods

n/a	Involved in the study
☒	☐ ChIP-seq
☒	☐ Flow cytometry
☒	☐ MRI-based neuroimaging

Antibodies

Antibodies used	CRX Abcam ab140603<br>ARR3 Proteintech Group Cat# 11100-2-AP, RRID:AB_2289959<br>EBF3 Abnova Corporation Cat# H00253738-M05, RRID:AB_565597<br>Ki-67 Abcam Cat# ab15580, RRID:AB_443209<br>TFF1 Sigma-Aldrich Cat# HPA003425, RRID:AB_1848260
Validation	All used antibodies were tested by the manufacturers for IHC: anti-CRX (Abcam, ab140603; https://www.abcam.com/crx-antibody-epr9582-ab140603.html), anti-ARR3 (Proteintech Group, 11100-2-AP; https://www.ptglab.com/products/ARR3-Antibody-11100-2-AP.htm), anti-EBF3 (Abnova Corporation, H00253738-M05; https://www.abnova.com/products/products_detail.asp?Catalog_id=H00253738-M05), anti-Ki67 (Abcam, ab15580; https://www.abcam.com/ki67-antibody-ab15580.html), and anti-TFF1 (Sigma-Aldrich, HPA003425; https://www.sigmaaldrich.com/FR/fr/product/sigma/hpa003425). Examples of human tissue IHC performed with anti-ARR3, anti-EBF3, anti-Ki67, and anti-TFF1 can be found in the literature and public resources: anti-ARR3 (PMID: 32971811, PMID: 30832308), anti-EBF3 (PMID: 30718509), anti-Ki67 (PMID: 32327630), and anti-TFF1 (Human Protein Atlas: https://www.proteinatlas.org/ENSG00000160182-TFF1/antibody#immunohistochemistry).

Eukaryotic cell lines

Policy information about cell lines

Cell line source(s)	All experiments with retinal organoids were carried out using the hiPSC-2 cell line, previously established by the authors from human dermal fibroblasts obtained by Dr. Rustin's laboratory (INSERM U676, Paris, France) (Reichman et al., 2014).
Authentication	Cell line is not authenticated but the characterization of the hiPSC-2 cell line consisted of positive alkaline phosphatase staining, immunohistochemistry, qRT-PCR analysis of pluripotency markers (NANOG, TRA1-81, OCT4, and SSEA4), capacity for embryoid body formation, differentiation towards the three main germ layers markers (endoderm, SOX17, mesoderm, BRACHYURY, SMA, and ectoderm PAX6, TUJ1), teratome formation in NSG mouse and karyotype analysis (Reichman et al., 2014).
Mycoplasma contamination	Absence of mycoplasma contamination was verified by theMycoAlert™Mycoplasma Detection Kit (selective biochemical test of mycoplasma enzymes) used according to the manufacturer's instructions (Lonza).
Commonly misidentified lines<br>(See ICLAC register)	No commonly misidentified cell lines used.

Human research participants

Policy information about studies involving human research participants

Population characteristics	Initial series of 102 retinoblastomas<br>We included 102 tumors from 50 male patients and 52 female patients in this study. These patients came from three different hospitals: the Institut Curie in Paris, France (78 patients), the Garrahan Hospital in Buenos Aires, Argentina (19 patients), and the Sant Joan de Déu Hospital in Barcelona, Spain (5 patients). Median age at diagnosis was 19.9 months (minimum: 27 days, maximum: 9.65 years). Six patients had received chemotherapy and/or radiotherapy prior to enucleation.<br><br>Series of 112 retinoblastomas with HRPFs<br>We included an independent series of 112 patients with high-risk pathological features (HRPFs) from the Garrahan Hospital (Argentina) in this study (61 females and 51 males). Median age at diagnosis was 31 months (range: 1 to 168 months). Among the 112 patients, 19 (9 females and 10 males) subsequently developed metastatic disease. The median time from retinoblastoma diagnosis to metastasis was nine months (range: 4 to 65 months). Additional clinical characteristics are included in Table 2 and Supplementary Table 6.<br><br>Additional retinoblastoma sample for single-cell RNA sequencing<br>One additional sample was studied at the single-cell level. The sample for single-cell RNA-seq was obtained from an enucleated patient > 18 months of age with a unilateral non-hereditary form of retinoblastoma who did not receive treatment prior to enucleation.<br><br>Fetal retina<br>Fetal retinas were obtained from legally-induced terminations of pregnancy. They were provided by the Fetal Pathology Unit of Antoine-Béclère Hospital in Paris (France). Three fetal retinas — RET215 (from a 20-week-old fetus), RET2 (23-week-old fetus), and RET1 (27-week-old fetus) were included in this study.
Recruitment	For the initial retrospective series of 102 retinoblastoma, patients were randomly recruited from the Institut Curie, Garrahan Hospital, and Sant Joan de Deu Hospital focusing mainly on patients without prior treatment before enucleation (n=96 without treatment, n=6 with treatment). Enucleations were performed at the Institut Curie between 1997 and 2007, at the Garrahan Hospital between 2011 and 2014, and at the Sant Joan de Déu Hospital between 2013 and 2014. For the retrospective series of 112 retinoblastoma cases with HRPFs (High-Risk Pathological Features) from the Garrahan Hospital, 19 metastatic cases were included. All available metastatic cases between January 1991 and October 2016 were selected and the remaining non-metastatic cases were randomly recruited among patients enucleated between January 2004 and December 2016.
Ethics oversight	This study was performed retrospectively and in accordance with the Declaration of Helsinki and the legislation of each participating country - France, Argentina, and Spain. The study was approved by the Institut Curie Review Board, the institutional review board of the hospital de Pediatria Juan P Garrahan and the Clinical Research Ethics Committee of Sant Joan de Déu Hospital. Written informed consent was obtained from the parents or legal guardians of retinoblastoma patients in accordance with current guidelines and legislation of each participating country.<br>Human fetuses (20, 23, 27 GW) were obtained from legally-induced terminations of pregnancy performed at the Antoine Béclère Hospital in France. Fetal tissues were collected with the women's written consent, in accordance with the legal procedure agreed by the French National Agency for Biomedical Research (Agence de Biomédecine) and the approval of the local ethics committee of the Antoine Béclère Hospital.

Note that full information on the approval of the study protocol must also be provided in the manuscript.
